# The impact of donor-specific antibodies’ presence on the outcome post-allogeneic hematopoietic stem cell transplantation: a survey from a single center

**DOI:** 10.3389/fonc.2024.1387181

**Published:** 2024-08-21

**Authors:** Simona Sica, Elisabetta Metafuni, Filippo Frioni, Maria Assunta Limongiello, Eugenio Galli, Federica Sorà, Andrea Bacigalupo, Elvira Poggi, Mariano Antonio Feccia, Annarita Manfreda, Patrizia Chiusolo, Sabrina Giammarco

**Affiliations:** ^1^ Dipartimento di Scienze di Laboratorio ed Infettivologiche, Fondazione Policlinico Universitario A. Gemelli IRCCS, Rome, Italy; ^2^ Sezione di Ematologia, Dipartimento di Scienze Radiologiche ed Ematologiche, Università Cattolica del Sacro Cuore, Rome, Italy; ^3^ CNR-IFT Roma San Camillo, Rome, Italy; ^4^ Centro Regionale Trapianti Lazio, Roma San Camillo, Rome, Italy

**Keywords:** donor-specific antibodies, primary graft failure, neutrophil and platelets engraftment failure, anti HLA antibodies, allogeneic stem cell transplantation (allo-SCT)

## Abstract

**Introduction:**

Donor-specific antibodies (DSAs) correspond to anti-HLA antibodies of the recipient that are specifically directed to a mismatched antigen of the donor. In the setting of solid organ transplantation DSAs are associated with rejection. Their role is still debated in allogeneic cell transplantation. International guidelines recommend testing patients for DSA before transplant, and if possible, choosing a donor with negative screening.

**Methods:**

We collected clinical data of 236 recipients of alloSCT, performed at our institution from March 2019 to October 2023, to evaluate their impact on engraftment. Serum from all patients was tested for DSA.

**Results:**

186 patients (79%) achieved sustained myeloid engraftment within day 30 post alloSCT. Thirty-two out 236 (13%) patients engrafted after day 30 post alloSCT. The median times to neutrophil engraftment and platelet engraftment were respectively 21 days (range 11-121 days) and 19 days (range 10-203 days). Fourteen out 236 patients (6%) experienced PrGF. .Twenty-nine patients (12 %) were DSA-positive. Among 29 patients with DSA positivity, 17 had a haploidentical donor and 12 had a UD donor. DSA positivity directly correlates respectively with neutrophil and platelets engraftment failure at 30 days after alloSCT (p=0.01 and p= 0.0004). Univariate Cox analysis showed that factors, including DSAs positivity, disease type, disease status, donor type, conditioning regimen, patient's age, and CD34+ were correlated with neutrophil and platelet engraftment failure at 30 days after alloSCT. Younger patients with DSA negativity, with acute leukemia, in complete response at the time of transplant, who received a higher dose of CD34+ cells from a sibling donor after a myeloablative conditioning regimen, have a reduced risk of neutrophil and platelet engraftment failure at day +30 post alloSCT.Multivariate analysis confirmed the impact of the presence of DSA only for platelet engraftment, confirming the role of type and status disease, donor type, recipient age, and CD34+ cells infused on engraftment. DSA presence has no impact on TRM, DFS, and OS.

**Discussion:**

PrGF has a multifactorial pathogenesis, where DSA is not the only player, but its impact could vary depending on the transplant platform. Thus patient screening may be helpful to choose the best donor and transplant strategy.

## Introduction

Anti-human leukocyte antigen antibodies (HLAs) are directed against class I (HLA-A, -B, -C) and or class II (HLA-DRB1, -DQB1, DP) HLA antigens, resulting from a previous exposure to non-self HLA antigens; risk factors for anti-HLA antibodies are represented by pregnancy, blood product transfusions (higher risk from receiving leukocyte and platelet transfusion rather than erythrocyte transfusions), prior organ transplantations, and gender, with a higher prevalence in female (86% vs 5%) (1).

Anti-HLA antibody detection has no clinical significance for a patient submitted to transplantation (2), while the donor-specific anti-HLA antibodies (DSAs), which are preformed antibodies of the recipient and specific for HLA antigens of the donor, are associated with an increasing risk of rejection in solid organ transplantation settings (3, 4)**;** their role in HCST is still debated (5).

Rejection is a potentially fatal complication, which consists in primary graft failure (PrGF). PrGF is a lack of engraftment of donor stem cells, consisting of cytopenia associated with a lack of donor chimerism on the bone marrow (6). PrGF is different from poor graft function, where the inadequate function of engrafted donor stem cells produces cytopenia, despite full donor chimerism (7).

Probably DSAs can cause graft failure by cytotoxicity, mediated by both antibodies and by complement, but the exact mechanism is not fully understood (8).

Other common risk factors for primary graft failure are represented by the disease, the remission status at transplantation time, the conditioning regimen intensity, HLA-mismatched graft, stem cell dose, stem cell source, AB0 incompatibility, and T-cell depletion (9).

In recent years, ELISA-based methods, flow cytometry, and Luminex platform are the most used tests for DSA screening, because they are more sensitive than the complement-dependent cytotoxicity (CDC) assay (10). The introduction of flow cytometry crossmatching gives the possibility of detection and quantifying antibody specificities.

## Materials and methods

The purpose of our study is to evaluate the impact of DSA presence on the outcome post-transplant, focusing our attention on engraftment. In this aim, in our retrospective study, we analyzed a heterogeneous population of 236 patients undergoing HCT. We tested DSA recipients of allogeneic SCT performed at our institution from March 2019 to October 2023. Patients’ characteristics are summarized in **Table 1**. There were 97 female patients and 139 male patients.

**Table 1:** Patients’ characteristics.

**Patients (n)**	**236**
**Median age (range)**	56 years (range 19–74 years)
**Gender**	M/F = 139/97
**Diagnosis** − Acute leukemia/MDS − Lymphoproliferative disease − Chronic myeloproliferative disease − Aplasia	− 148 (63%) − 24 (10%) − 52 (22%) − 12 (5%)
**Disease status before HCT** − Complete remission − Stable disease − Progressive disease − HCST frontline	− 114 (48%) − 97 (41%) − 23 (10%) − 4 (1%)
**Conditioning regimen** − Myeloablative − Reduced intensity − CTX	− 153 (64%) − 83 (35%) − 1 (4%)
**Donor** − MUD 7/8 − MUD 8/8 − SIB − HAPLO − CB	− 41 (17%) − 86 (36%) − 38 (16%) − 68 (30%) − 3 (1%)
**GvHD prophylaxis** − CSA, CTX, MMF − CSA, CTX, MTX, ATG	− 232 (98%) − 4 (2%)
**CD34+ cell median (range)**	5.9 × 10^6^/kg (1.6–15)
**Acute GvHD grade >/=2**	57 (24%)
**Chronic GvHD grade >/=2**	23 (10%)
**Presence of anti HLA antibodies**	29 (12%)

CTX, cyclophosphamide; CSA, cyclosporine; MMF, mycophenolic acid; MTX, methotrexate; ATG, anti-thymocyte globulin.

The median age was 56 years (range 19–74). HCT was performed for acute leukemia and myelodysplastic syndrome in 148 patients (63%), chronic myeloproliferative disease (myelofibrosis or chronic myeloid leukemia) in 52 patients (22%), severe aplastic anemia in 12 patients (5%), and lymphoproliferative disorders in 24 patients (10%) (**Table 1** **).**

At the time of transplant, 114 patients (48%) were in complete remission, 97 (41%) were in stable disease, and 23 patients (10%) had a progressive disease; two (1%) patients underwent HSCT as frontline therapy.

There were 153 patients (64%) who underwent a myeloablative conditioning regimen with the association of thiotepa (5 mg/kg on days −6 and −5), fludarabine (50 mg/m^2^ once a day iv on days −4, −3 and −2), and busulfan (0.8 mg/kg four times a day on days −4, −3, and −2) (TBF); a reduced intensity, a conditioning regimen with the Baltimore scheme, including cyclophosphamide 14.5 mg/kg on days −6 and −5 and fludarabine 50 mg/kg on days −6, −5, −4, −3, and −2, and total body irradiation with 2 Gray on day −1 (11) were performed in 83 patients (35%), and only cyclophosphamide (50 mg/kg for 4 days) was used in 4 patients (1%).

The main GvHD prophylaxis scheme consisted of cyclosporine (CSA), mycophenolic acid (MMF), and post-transplant cyclophosphamide (PTCY), used in 232 patients (98%). Only four patients (2%) received CSA, anti-thymocyte globulin, and methotrexate.

There were 38 donors who were identical siblings (16%), 68 were haploidentical family donors (30%), 127 were unrelated donors (54%), 86 (68%) were matched unrelated donors (8/8), and 41 (32%) were mismatched unrelated donors (7/8).

Patients received a median dose of CD34+ stem cells of 5.9 × 10^6^/kg (range 1.6 × 10^6^/kg–15 × 10^6^/kg). The stem cell source was predominantly peripheral blood (225 patients, 95%). Eight patients received bone marrow stem cells and three patients a cord blood unit.

All patients were screened for the presence of DSAs (class IgG and IgM) with a FlowPRA Screening Test and, only if tested positive, were quantitatively determined with Luminex Single Antigen Beads. The test is based on a series of polystyrene microspheres (beads). Every beads contain fluorochromes of differing intensity embedded within the bead, giving each group of beads with an HLA molecule or molecules derived from lymphoblastoid cell lines attached with a unique signal. The Luminex platform has a greater sensitivity than ELISA and nowadays represents the gold standard for DSA testing. A positive result was considered a median fluorescence intensity (MFI) cutoff >1,000 (10).

Primary graft failure (PrGF) represents the lack of blood count recovery by day +28, with donor chimerism <10%. All patients were tested for chimerism on bone marrow cells at day +30 after HCT, by PCR analysis of highly polymorphic short tandem repeats (STR). Comparing the STR expression profiles between recipient, donor, and post-transplant samples, we can obtain donor chimerism percentage, according to international guidelines (12).

### Statistical analysis

Statistical analysis was performed using NCSS 19 Statistical Software−2016 (NCSS, LLC, Kaysville, Utah, USA; ncss.com/software/ncss). Data are shown as median and range for continuous variables and as number and proportion for categorical variables. The cumulative incidence of neutrophils, platelets, and reticulocyte engraftment was calculated at day 30 according to DSA presence. Comparison between curves was made using the Grey’s test. Variables affecting the time-dependent outcome such as platelets, neutrophils, and reticulocyte engraftment were identified by the Cox regression method. Statistical significance was attributed for p-value <0.05. In multivariate analysis were included only variables with a p-value ≤0.05 in univariate analysis.

The Kaplan–Meier method was used for overall survival (OS), and comparison between curves was assessed with a log-rank test.

## Results

Neutrophil engraftment (neutrophil count ≥0.5 × 10^9^/L) within day +30 after HCT was reached in 186 patients (79%) at a median interval from the transplant of 21 days (range 11–121). The median time to platelet engraftment (platelet count ≥20 × 10^9^/L) was 19 days (range 10–203 days).

Primary graft failure (PrGF) was seen in 14/236 patients (6%) with failure to recover a neutrophil count ≥0.5 × 10^9^/L: it occurred in one patient after TBF3 (3 days of busulfan), three patients after TBF2 (two days of busulfan), seven patients after TBF1 (one day of busulfan), and three after the Baltimore conditioning regimen. We found 4 patients with PrGF in the group with DSA (13%) and 10 patients in the group with no DSA (4.8%) (p = 0.06).

There were 29 patients (12%) who were DSA-positive. A total of 15 patients had antibodies against HLA class I antigens, 10 patients had antibodies against HLA class II, and 4 patients had antibodies against classes I and II. Among 29 patients with DSA positivity, 17 patients received graft from a haploidentical donor, seven patients from an MUD, and five patients from an MMUD. Among patients who experienced PGF, the four patients with DSA received graft from a haploidentical donor.

Acute and chronic GVHDs were present respectively in 57/236 (24%) and 23/236 (10%) patients.

We found that DSA positivity directly correlates with neutrophil and platelet engraftment failure at 30 days after allogeneic SCT (p = 0.01; p = 0.0004). We found no correlation between DSA presence and reticulocyte engraftment.

**Figure 1:**
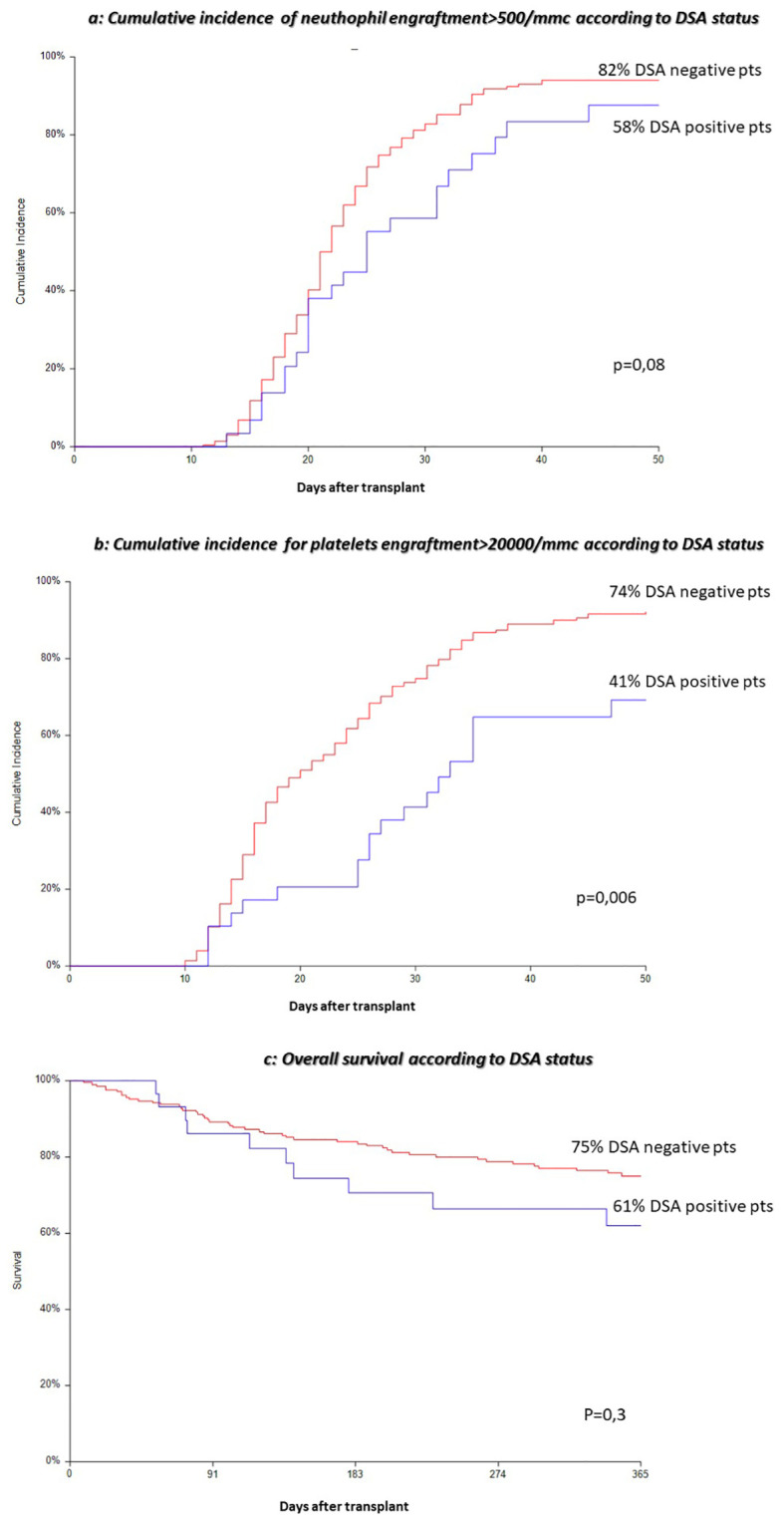
**(A)** Cumulative incidence of neutrophil engraftment>500/mmc according to DSA status. **(B)** Cumulative incidence for platelets engraftment>20000/mmc according to DSA status. **(C)** Overall survival according to DSA status.

Cumulative incidence of neutrophil engraftment at day +30 post alloSCT resulted in 58% in the DSA+ group versus 82% in the DSA− group (p = 0.08) (**Figure 1A**).

Cumulative incidence of platelet engraftment at day +30 post alloSCT resulted in 41% in the DSA+ group versus 74% in the DSA− group (p = 0.006) (**Figure 1B**).

Cumulative incidence of reticulocyte engraftment at day +30 post alloSCT resulted in 17% in the DSA+ group versus 34% in the DSA− group (p = 0.07).

The median MFI at transplant was 5,933 with a range value between 2,000 and 23,769, and we found a correlation between PrGF and degree of MFI intensity (p = 0.01). The four patients with DSA who experienced PrGF have a higher MFI with a median value of 11,547 (range 2,000–23,769), compared with patients with DSA without PrGF with a median value of 4,500 (range 2,000–21,530). We can suppose that patients with an MFI value greater than 10,000 could be at risk of PGF.

Univariate Cox analysis was performed to evaluate which factors could influence engraftment after allogeneic SCT. This analysis showed that factors, including DSA positivity (p = 0.02), disease type (p = 0.0001), disease status (p < 0.0001), donor type (p = 0.0001), conditioning regimen (p = 0.04), age at transplant (p = 0.003), and number of CD34+ cells infused (p = 0.0003) were correlated with neutrophil engraftment failure at 30 days after alloSCT (**Table 2A**). The same factors have the same impact on platelet engraftment, as reported in **Table 2B**.

**Table 2A:** Univariate and multivariate Cox analyses: neutrophil engraftment at day 30 post-HCT.

Risk factors	Univariate		Multivariate	
	Hazard ratio (95% CI)	p	Hazard ratio (95% CI)	p
**DSA** pos vs neg	0.52 (0.28-0.95)	**0.02**	0.65 (0.37-1.13)	0.12
**Disease** *MFI vs AL*	0.36 (0.23-0.57)	**<0.0001**	0.48 (0.27-0.86)	**0.01**
**Conditioning** *RIC vs ABL*	0.73 (0.53-0.99)	**0.04**	0.87 (0.61-1.23)	0.44
**Stem cell source** *PBSC vs others*	0.7 (0.45-1.34)	0.3	–	–
**Disease status at transplant** *No CR vs CR*	0.44 (0.33-0.60)	**<0.0001**	0.47 (0.31-0.71)	**0.0004**
**Donor** *SIB vs UD* *SIB vs Haplo*	0.57 (0.39-0.85) 0.40 (0.25-0.62)	**0.005** **0.0001**	0.6 (0.39-0.90) 0.46 (0.28-0.75)	**0.01** **0.001**
**CD34+ cell** continuous	1.11 (1.04-1.17)	**0.0003**	1.12 (1.06-1.19)	**P<0.0001**
**Age** *continuous*	0.98 (0.97-0.99)	**0.003**	0.99 (0.97-1.00)	0.10
**Recipient sex** *Female vs male*	0.98 (0.62-1.39)	0.7	**-**	**-**
**GvHD prophylaxis** PTCY vs others	0.55 (0.20-1.34)	0.24	**-**	**-**

MFI, myelofibrosis; AL, acute leukemia; RIC, reduced intensity conditioning regimen; ABL, myeloablative conditioning regimen; PBSC, peripheral blood stem cells; SIB, sibling donor; UD, unrelated donor; HAPLO, haploidentical donor.

Risk factors affecting neutrophil engraftment failure at 30 days after HCT: DSA positivity (p = 0.02), disease type (p = 0.01), disease status (p < 0.0001), donor type (p= 0.0001), conditioning regimen (p = 0.04), age at transplant (p =0.003). Multivariate analysis confirmed the role of disease type (p <0.0001), disease status (p = 0.0004), donor type (p= 0.001), and number of CD34+ cells infused (p <0.0001).

**Table 2B:** Univariate and multivariate Cox analyses: platelet engraftment at day 30 post-HCT.

Risk factors	Univariate		Multivariate	
	Hazard ratio (95% CI)	p	Hazard ratio (95% CI)	p
**DSA** pos vs neg	0.40 (0.22-0.72)	**0.02**	0.43 (0.23-0.82)	**0.01**
**Disease** MFI vs AL	0.52 (0.32-0.82)	**0.005**	0.62 (0.34-1.11)	0.11
**Conditioning** RIC vs ABL	0.7 (0.50-0.97)	**0.03**	0.89 (0.56-1.41)	0.6
**Stem cell source** PBSC vs others	0.52 (0.19-1.40)	0.19	–	–
**Disease status at transplant** no CR vs CR	0.52 (0.42-0.77)	**0.0004**	0.58 (0.38-0.90)	**0.01**
**Donor** SIB vs UD SIB vs Haplo	0.66 (0.38-1.17) 0.4 (0.27-0.71)	0.1 **0.0009**	1.10 (0.71-1.70) 0.70 (0.42-1.16)	0.65 0.17
**CD34+ cell** continuous	1.14 1.076-1.20)	**<0.0001**	1.17 (1.10-1.24)	**<0.0001**
**Age** continuous	0.9 (0.5-0.9)	**<0.0001**	0.97 (0.96-0.99)	**<0.0001**
**Recipient sex** Female vs male	0.93 (0.62-1.39)	0.7	**-**	**-**
**GvHD prophylaxis** PTCY vs others	0.78 (0.25-2.46)	0.67	**-**	**-**

MFI, myelofibrosis; AL, acute leukemia; RIC, reduced intensity conditioning regimen; ABL, myeloablative conditioning regimen; PBSC, peripheral blood stem cells; SIB, sibling donor; UD, unrelated donor; HAPLO, haploidentical donor.

Risk factors affecting platelets engraftment failure at 30 days after HCT: DSA positivity (p = 0.02), disease type (p =0.005), disease status (p=0.0004), donor type (p= 0.0009), conditioning regimen (p = 0.03), age at transplant (p <0.0001), and number of CD34+ cells infused (p < 0.0001). Multivariate analysis confirmed the role of DSA positivity (p=0.01), disease status (p=0.01) age (p < 0.0001) and number of CD34+ cells infused (p < 0.0001).

Multivariate analysis confirmed a role for the presence of DSA only in the setting of platelet engraftment [HR 0.43 (95% CI 0.23–0.82), (p = 0.01)]. Age at transplant, disease type, disease status at transplant, donor type, and number of CD34+ cells infused remain strong factors affecting trilinear recovery after SCT (**Tables 2A, B**).

One-year overall survival was 70%. Causes of death were disease relapse in 35 patients, chronic Graft versus host disease (GVHD) in 10 patients, and transplant-related mortality (TRM) in 25 patients.

DSA presence has no impact on TRM, relapse, disease-free survival (DFS), and OS (**Figure 1C**).

Among the 14 patients who experienced PrGF, seven (50%) patients received a second transplant. In two patients, the donor was the same, and in the remaining five patients, the donor was different. The stem cell source was peripheral blood. The conditioning regimen was performed according to the Baltimore scheme, where the TBI was replaced with melphalan at a dosage of 30 mg/kg, due to the severe cytopenia of these patients, except for one patient, who was treated with a low dose of fludarabine and cyclophosphamide. Patients’ characteristics are summarized in **Tables 3A**, **B**.

**Table 3A:** Clinical characteristics of patients with primary graft failure at first alloSCT.

n	Patient gender	Age	DX	Disease status at 1 SCT	Conditioning regimen	Donor gender	Donor age	SC Source	CD34+ cell dose	DSA
**1**	F	64	AML	CR1	TBF2	M	38	BM	3.7	POS
**2**	M	69	AML	FRONTLINE	TBF1	M	41	BM	4.2	NEG
**3**	M	69	AML	FRONTLINE	TBF1	F	33	BM	6.8	POS
**4**	M	73	MFI	STABLE	TBF1	M	46	BM	3.8	NEG
**5**	F	60	MDS	CR1	TBF2	M	30	BM	7.5	POS
**6**	M	43	AML	CR1	TBF3	M	0	CBU	2.5	NEG
**7**	M	69	MDS	STABLE	TBF1	F	30	PBSC	5.8	POS

AML, acute myeloid leukemia; MFI, myelofibrosis; MDS, myelodysplastic syndrome; CR1, first complete remission; TBF3, 3 days of busulfan; TBF2, 2 days of busulfan; TBF1, 1 day of busulfan; BM, bone marrow; PBSC, peripheral blood stem cell; CBU, cord blood unit.

**Table 3B:** Clinical data of seven patients at second alloSCT.

N	Donor 2^nd^ Tx	Cond 1^st^ 2^nd^	Int-dd 2^nd^ Y/N	Engr	GvHD grade	Alive 1 year Y/N	Cause death
1	same	MEL	42	Y	III	N	GvHD
2	other	MEL	39	No	0	Y	–
3	other	Flu-CTX	48	Y	0	Y	–
4	other	MEL	53	Y	III	Y	–
5	same	MEL	32	Y	I	Y	–
6	other	MEL	30	No	0	N	Sepsis
7	other	MEL	58	Y	0	Y	–

Donor 2^nd^ Tx, donor of the second transplant; same, the same HAPLO donor as in the first transplant; other, other HAPLO family member; Int-dd 1^st^–2^nd^, interval in days between the first and second HAPLO transplants; Engr 2^nd^, engraftment after a second HAPLO Yes/NO; aGvHD, acute GvHD grade.

Two of these patients were also submitted to a desensitization treatment before second transplant, in order to reduce DSA titer, with plasmapheresis and donor platelet infusion. In this way, we reduced the median MFI title from 24,874 to 4,500. After the second transplant, five out seven patients are alive (71%), with one patient who died because of gut GVHD after engraftment and one patient because of sepsis.

## Discussion

Donor-specific antibodies (DSAs) correspond to anti-HLA antibodies, which are specifically direct against a mismatched antigen of the donor (5). Healthy non-transplanted individuals can have DSA as a result of common exposures including pregnancy; on the other hand, patients can present DSA due to blood product transfusion, or as a result of previous organ transplantation (13). DSA presence at the time of transplant is correlated with graft rejection and decreased survival in solid organ transplantation (14–16). The American Society for Histocompatibility and Immunogenetics (ASHI) strongly recommends DSA tests as mandatory in solid organ transplants.

DSA seems to have an important role as a barrier against successful engraftment of donor cells, with a negative impact on survival, especially in the setting of haploidentical stem cell transplant. Their role appears to be more prevalent in this setting due to the degree of mismatch and to a higher likelihood of alloimmunization of multiparous females against offspring’s HLA antigens (17).

Previous studies reported the association between DSA and PrGF in HSCT with unrelated HLA mismatched donors or CBU.

Takanashi et al. showed that the graft failure rate was significantly higher with a lower neutrophil recovery in the DSA-positive patients, compared with the negative ones, in unrelated cord blood transplantations (68% vs 17%) (18).

Cutler et al. reported similar data in the setting of double UCB HCT, with a graft failure rate of 57% in the DSA-positive patients vs. 6% in the DSA-negative patients, respectively (19).

Spellman et al. showed that 24% of patients with PrGF had performed DSA in the setting of unrelated donors (20). Similarly, Ciurea et al. found that the presence of DSA was the only factor associated with a significantly higher rate of graft failure, in the MUD transplant population (DSA+ 38% vs DSA− 3%) (21).

The same author in a largest study confirmed the same finding in the setting of haploidentical HCT: in a 122-patient population, the incidence of DSA was 18% and 32% of the patients with DSA experienced PrGF, while only 4% of patients without DSAs had PrGF (p < 0.001). DSAs’ presence has a significant impact also on delayed engraftment (19 days vs. 18 days, p = 0.004) (22).

In another study on 79 patients submitting to haploidentical HCT, Yoshihara et al. found a significantly lower cumulative incidence of donor neutrophil (61.9% vs. 94.4%, p = 0.026) and platelet engraftment (28.6% vs. 79.6%, p = 0.035) in the DSA-positive group (23).

Moreover, Chang et al. showed that DSA positivity was associated with PrGF and delayed engraftment but also with poor graft function (5).

In our study on 236 patients, we focused our attention on the impact of DSA presence on the outcome post-transplant, in particular on engraftment. There were 29 patients (12%) who showed DSA positivity. We found that DSA positivity directly correlates with neutrophil and platelet engraftment failure at 30 days after allogeneic SCT (p = 0.01) (p = 0.0004), but we did not find a direct correlation with PrGF.

Univariate Cox analysis showed that younger patients with DSA negativity, with acute leukemia, who had an incomplete response at the time of transplant, and who received a higher dose of CD34+ from a sibling donor after a myeloablative conditioning regimen have a reduced risk of neutrophil and platelet engraftment failure at day +30 post alloSCT. In multivariate analysis, the presence of DSAs continued to be significantly associated only with platelet engraftment delay: [RR 0.43 (95% CI 0.23–0.82) (p = 0.01)]. Thus, we confirmed an association between DSA presence and a delay of engraftment, but not with PrGF. In our population, DSA’s presence has no impact on TRM, relapse, DFS, and OS. With the limit of a retrospective study, on a population of 236 patients, the incidence of DSA positivity is similar to that reported in literature, and, as previously described, DSA may have a role in the engraftment, but it is not the only potential risk factor and its impact may depend on the sum of the other factors.

Other studies found no correlation between DSA presence and impact on PrGF, as reported by Altared et al. in a population of 107 patients submitted to haploidentical transplant, after receiving a myeloablative conditioning regimen: there is no evidence of DSA in the three patients who experienced graft failure. On the other hand, engraftment was achieved in the 17 DSA-positive patients, similarly to 87 DSA-negative patients (24).

Moreover, DSA seems to have no role on PrGF, and neither in predicting engraftment after a second HAPLO graft, as reported in another study (25, 26).

The host immunologic reaction against donor cells is the principal cause of graft rejection. Graft rejection is due to cytolytic reaction mediated by T and/or NK cells of the recipient, who survived the conditioning regimen. The immunologic reaction is based on the genetic disparities between the donor and the recipient and the status of anti-donor alloreactivity of the recipient. This is more likely to happen in mismatched unrelated and haploidentical donors (13).

On the other hand, in more recent years, *in vitro* and *in vivo* studies have demonstrated that there is also an antibody-mediated graft rejection humoral, called humoral rejection (27).

The humoral rejection can occur by cytotoxicity mediated by antibodies and by complement. An MFI above 1,000 is indicative of a positive screening for DSA. As reported in several studies, the incidence of PGF appears to be directly correlated with MFI levels above 5,000 (22, 23). We confirmed these data also in our study, where we found a correlation between PrGF and degree of MFI intensity (p = 0.01). As reported by Ciurea et al., the risk of rejection rate for patients with DSA >5,000 MFI was significantly higher (54% vs 9%) (21). Furthermore, higher MFI levels (>5,000) also correlate with the complement-binding ability. It remains unclear whether this depends on the higher ability of some antibodies to bind complement or on the higher levels of antibodies, which are more likely to activate complement cascade and destroy targeted cells. On the other hand, Chen et al. found no correlation between MFI levels and the ability to fix complement (28). Due to these discrepant data, a uniform MFI cutoff cannot be used as a surrogate for clinical relevance.

As suggested by EBMT guidelines, all patients should be screened before transplant, to select donors with no DSA against, when possible, and desensitize patients with DSA before transplant (17).

There are four strategies to desensitize patients with DSA. The first one consisted in mechanical antibody removal by using plasmapheresis or immunoabsorption. The second one is based on the use of monoclonal antibodies against CD20+ B lymphocytes (rituximab), and proteasome inhibitors against plasma cells, to inhibit the antibody production. The third procedure is based on the antibody neutralization by infusion of intravenous immunoglobulin (IVIg), and platelet or buffy coat transfusions. Another strategy is the inhibition of complement cascade, by using anti C5 or C3 agents (17, 18).

Among the seven patients with PrGF, who were submitted to a second alloHCT, four patients resulted positive for DSA. Among these, two female patients resulted positive for DSA against their offspring donor, with a higher MFI. After the first transplant and before the second one, patients were submitted to desensitization therapy, which included plasmapheresis and platelet transfusion. In this way, we reduced the median MFI title from 24,874 to 4,500.

## Conclusion

The increasing use of haploidentical and mismatched unrelated donors highlighted new interest in the field of DSA. Their role in the setting of alloSCT is still debated, with conflicting data from several studies. Probably different results depend on the multifactorial pathogenesis of PrGF, where DSA presence is a potential risk factor, where its weight varies according to several transplant settings.

EBMT consensus guidelines recommend testing DSAs in all patients. Donors with a negative DSA screen should be selected. If this is not possible, it is recommended to reduce the antibody levels prior to transplant, in order to favor a successful engraftment.

## Data Availability

The raw data supporting the conclusions of this article will be made available by the authors, without undue reservation.
